# Oxidative stress-driven epigenetic reprogramming of immune cells in COPD: from epitranscriptomic and metabolic crosstalk to treatable traits

**DOI:** 10.3389/fimmu.2026.1865051

**Published:** 2026-08-18

**Authors:** Chun Feng Pan, Qiong Wan, Feng-Xian Ni, Pan Xu, Dong-Hui Huang, Ze-Bo Jiang

**Affiliations:** 1 Zhuhai Hospital of Integrated Traditional Chinese and Western Medicine, Zhuhai, Guangdong, China; 2 Zhuhai Hospital, Faculty of Chinese Medicine, Macau University of Science and Technology, Zhuhai, Guangdong, China

**Keywords:** COPD, epigenetic reprogramming, immunometabolism, m^6^A modification, NETosis, oxidative stress, treatable traits

## Abstract

Chronic obstructive pulmonary disease (COPD) is a heterogeneous syndrome characterized by persistent oxidative stress and maladaptive immune responses, rather than a single disease entity. Oxidative stress not only damages lung tissue but also reprograms immune cells through both classical epigenetic mechanisms (DNA methylation, histone modifications) and epitranscriptomic regulation (m^6^A RNA methylation), shaping disease endotypes and treatment resistance. This review presents an integrated framework in which redox signals dynamically reshape the epigenetic and epitranscriptomic landscape, thereby locking immune cells into pathogenic states. Metabolic intermediates (S-adenosylmethionine, α-ketoglutarate, succinate, NAD^+^) serve as critical nodes that connect immunometabolism to both classical epigenetic enzymes and the m^6^A machinery, thereby linking redox status to RNA fate. Using NETosis as a paradigm, we illustrate how oxidative–epigenetic–metabolic loops sustain neutrophilic inflammation and resolution failure. Finally, we outline a treatable traits framework that integrates these mechanistic insights into precision combination therapies. This conceptual roadmap aims to shift COPD management from symptom control toward durable, mechanism-driven disease modification.

## 1 Introduction

Chronic obstructive pulmonary disease (COPD) is increasingly recognized as a syndrome of marked heterogeneity, rather than a single disease entity (1, 2). Clinically, patients are classified into phenotypes—chronic bronchitis, emphysema-dominant disease, and asthma-COPD overlap (ACO)—based on symptom patterns, radiographic findings, and physiological measures (3). Immunologically, however, this phenotypic taxonomy conceals a deeper and more consequential diversity of endotypes, which are defined by distinct immune activation patterns—ranging from neutrophilic and eosinophilic to pauci-inflammatory profiles (4, 5). These endotypes determine disease trajectory, exacerbation susceptibility, and therapeutic responsiveness. Yet current standard-of-care therapies (bronchodilators, inhaled corticosteroids, phosphodiesterase-4 inhibitors) are largely phenotype-driven and do not target endotype-specific drivers (6). The result is an unsatisfactory landscape in which many patients fail to derive durable benefit.

A near-universal feature across all COPD endotypes is elevated oxidative stress (7). Driven by exogenous insults like cigarette smoke and endogenous inflammation, reactive oxygen and nitrogen species (ROS/RNS) impose a sustained burden on the airways and lung parenchyma (8–10). Oxidative stress has long been viewed as a direct cause of tissue injury through lipid peroxidation, protein carbonylation, DNA damage, and mitochondrial dysfunction (8, 11, 12). Beyond direct tissue damage, oxidative stress functions as a potent modulator of intracellular signaling networks (13). ROS/RNS alter kinase cascades, transcription factor activity, and redox-sensitive switches (14). This dual role—direct cytotoxicity plus signaling modulation—helps explain why oxidative stress associates with both progressive tissue destruction (emphysema) and chronic airway inflammation (15–17). But it does not explain why similar exposures give rise to distinct immune endotypes across patients.

We propose that epigenetic and epitranscriptomic context is the key variable that determines the cellular and immune system response to oxidative stress. Throughout this review, we use “epigenetic regulation” to refer collectively to DNA methylation, histone modifications, and non-coding RNA networks—mechanisms operating at the transcriptional level through chromatin-based regulation. We use “epitranscriptomic regulation” specifically for RNA modifications such as N^6^-methyladenosine (m^6^A), which operate post-transcriptional level by modulating mRNA stability, splicing, nuclear export, and translation. Both layers function as cellular memory, encoding past exposures and shaping future responses (18). DNA methylation, histone modifications (such as acetylation, methylation, and crotonylation), chromatin remodeling, non-coding RNAs (microRNAs, long non-coding RNAs), and RNA modifications (notably m^6^A) collectively govern transcriptional and post-transcriptional responsiveness in a cell type–specific manner (19–21). Because these modifications can be stable over long periods and are influenced by both genetic background and prior environmental exposures, they provide a mechanistic substrate for persistent phenotypic differences among individuals with similar ongoing exposures.

Crucially, oxidative stress directly modulates the enzymes that write, read, and erase epigenetic and epitranscriptomic marks (22). This places oxidative stress at the heart of a feed-forward circuit; redox changes alter regulatory enzymes, which then reshape immune and reparative gene programs—potentially locking cells into maladaptive states (23). For example, ROS inhibit histone deacetylase 2 (HDAC2), a key mediator of corticosteroid responses, leading to steroid resistance and exaggerated inflammation (24–26). ROS can activate DNA methyltransferases (DNMTs), promoting *de novo* DNA methylation patterns that silence anti-inflammatory or reparative genes (27–29). Conversely, the ten-eleven translocation (TET) family of DNA demethylases requires Fe(II) and α-ketoglutarate (α-KG), cofactors sensitive to redox and metabolic shifts; oxidative stress can thus perturb TET activity and lock in aberrant methylation patterns (30, 31). Similarly, m^6^A demethylases FTO and ALKBH5 are Fe(II)/α-KG-dependent dioxygenases whose catalytic function is sensitive to metabolic intermediates and redox balance (32, 33). Through such direct biochemical interactions, oxidative stress rewires the epigenetic machinery to produce heterogeneous, and often durable, alterations in cell identity and function.

Recasting COPD pathogenesis in terms of oxidative-epigenetic-epitranscriptomic reprogramming has important therapeutic implications (32, 34). It explains why simple antioxidant strategies have delivered only modest clinical benefit: neutralizing ROS removes an active trigger but does not erase the epigenetic “scars” deposited during prior redox insult. These scars persist, maintaining aberrant inflammatory programs even after oxidant burden is reduced. Thus, therapies targeting only oxidants are incomplete unless paired with strategies that reset epigenetic dysregulation.

This review provides an integrative framework connecting oxidative stress, epigenetic/epitranscriptomic modulation, and immunometabolism to immune cell dysfunction in COPD. We first map the oxidative-epigenetic circuitry across major immune and barrier cell types—alveolar macrophages, recruited monocytes, neutrophils, various T-cell subsets, and innate lymphoid cells. Second, we integrate immunometabolism and epitranscriptomics into this model. Third, we link oxidative-epigenetic dysfunction to key pathobiological processes such as NETosis and resolution failure, and briefly consider its emerging implications for trained immunity. Finally, we discuss the integration of these mechanistic insights into the treatable traits framework and outline future directions for precision therapy.

## 2 Oxidative stress in COPD: sources, targets, and immunological consequences

Oxidative stress is a central and persistent feature of COPD pathobiology (35). Within the lung, a sustained oxidant burden initiates molecular and cellular events that shape immune responses, impair resolution and promote structural damage (36, 37). This section maps the principal cellular sources of ROS/RNS, examines how distinct oxidant species determine their molecular targets, and details functional consequences for key immune cells.

### 2.1 Cellular sources and physicochemical diversity of ROS

The COPD lung harbors multiple ROS sources. Cigarette smoke delivers free radicals, redox-active chemicals (e.g., semiquinones, peroxides, nitric oxide), and metal catalysts (38, 39). Ambient particulate matter (PM_2.5_), ozone and nitrogen oxides contribute significantly (40–43). Endogenous sources include mitochondrial electron leakage (complexes I and III), NADPH oxidases (NOX2 in phagocytes, NOX4 in epithelial cells), xanthine oxidase and myeloperoxidase (44–47).

The biological impact of ROS depends critically on chemical identity and subcellular localization. Superoxide (O_2_^-^) is short-lived and rapidly dismutated to hydrogen peroxide (H_2_O_2_), which is more stable and membrane-permeant. The hydroxyl radical (•OH) indiscriminately damages macromolecules, while peroxynitrite (ONOO^-^) nitrates tyrosine residues (48). ROS generation is highly compartmentalized—within phagosomes, in mitochondrial cristae or at the plasma membrane—creating local high-concentration environments that selectively modify nearby proteins, lipids or nucleic acids (49). This spatial restriction means that bulk oxidant measurements are insufficient to predict biological outcomes.

### 2.2 Functional consequences for immune cells

Reactive species affect immune cell phenotype and function at multiple levels—signaling pathways, transcription factors, and oxidative post-translational modification (50–52). The cell-type-specific effects most relevant to COPD are discussed below.

Neutrophils are profoundly influenced by oxidative stress (53, 54). Neutrophil extracellular trap (NET) formation is tightly linked to NOX2-derived ROS, which facilitate chromatin decondensation and activate peptidylarginine deiminase 4 (PAD4) (55, 56). In COPD, neutrophils are primed toward exaggerated NETosis, contributing to airway obstruction, mucus plugging, and proteases release (57, 58). Jun Chen et al. reported that NETs-DNA promotes NF-κB-dependent autoimmunity via cGAS/TLR9 in long-term cigarette smoke exposure-induced COPD (57). NETs also provide a scaffold for oxidant-modified proteins that may become autoantigens (55). Beyond NETosis, ROS activate pro-survival signaling pathways including ERK and PI3K/Akt while inhibiting apoptotic pathways in neutrophils, thereby extending their lifespan in tissues and increasing local protease release and oxidative damage (56).

Macrophages and monocytes are also major targets of oxidative stress (59). Oxidative stress favors an M1-like polarization by stabilizing HIF-1α (60), while impairing M2-like pro-resolving phenotypes (61, 62). Efferocytosis is compromised by oxidative modification of receptors (63), prolonging inflammation. Oxidative modification of self-proteins creates neoepitopes that may promote autoreactive T-cell responses (64). In COPD alveolar macrophages, these changes result in reduced bacterial phagocytosis, impaired clearance of apoptotic cells, and persistent release of pro-inflammatory cytokines such as IL-6, TNF-α, and CXCL8 (65).

T lymphocytes are highly sensitive to redox balance (66). Regulatory T cells (Tregs) are particularly vulnerable: ROS reduce FOXP3 expression and impair Treg suppressive capacity (67). Oxidative environments favor Th17 differentiation via HIF-1α and STAT3 (68). Researchers have shown that T helper (Th) 17/regulatory T (Treg) imbalance is involved in COPD (69). Oxidative stress also alters CD8^+^ T-cell function, promoting senescence-like phenotypes (70).

Dendritic cells and B cells have gained increasing recognition in COPD immunopathology and deserve separate discussion. Dendritic cells (DCs) are the central antigen-presenting cells linking innate and adaptive immunity. In COPD, cigarette smoke exposure impairs DC maturation and antigen-presenting function (71–74). Smoke-exposed DCs show reduced expression of MHC class II and co-stimulatory molecules (CD80, CD86), compromising their ability to prime protective T-cell responses. Simultaneously, these DCs produce IL-6 and IL-23, promoting Th17 skewing (73). Oxidative stress also induces the accumulation of inflammatory CD11b^+^ DCs in the COPD lung, which produce TNF-α and IL-1β (75, 76). Importantly, oxidative modification of self-proteins enhances their uptake by DCs and presentation to T cells, potentially breaking self-tolerance (77, 78). This mechanism may explain the emergence of autoimmune features in subsets of COPD patients. B cells have gained recognition in COPD pathogenesis. Tertiary lymphoid follicles containing B cells, plasma cells, and T cells are found in the lungs of patients with severe COPD (79, 80). Oxidative stress promotes B-cell survival and activation through NF-κB and BAFF pathways (81). B cells in COPD produce autoantibodies against citrullinated proteins, oxidized phospholipids, and other modified self-antigens (78). The presence of anti-citrullinated protein antibodies (ACPAs) correlates with emphysema severity and faster lung function decline (79). B-cell-derived IL-6 and TNF-α contribute to local inflammation, while immune complexes activate macrophages and neutrophils via Fc receptors (82). These findings position B cells as both effector cells and potential therapeutic targets in COPD.

Innate lymphoid cells (ILCs), including ILC2s and ILC3s, are responsive to oxidative and metabolic cues (83); ROS may push ILC plasticity toward pro-inflammatory phenotypes that support type-2 or type-3 cytokine environments, thereby influencing eosinophilic or neutrophilic pathways (84). Airway epithelial cells suffer impairs barrier function and mucociliary clearance, promoting mucous metaplasia (85). Endothelial dysfunction contributes to vascular leak and tissue hypoxia, amplifying oxidative signaling via HIF pathways (86).

**Figure 1:**
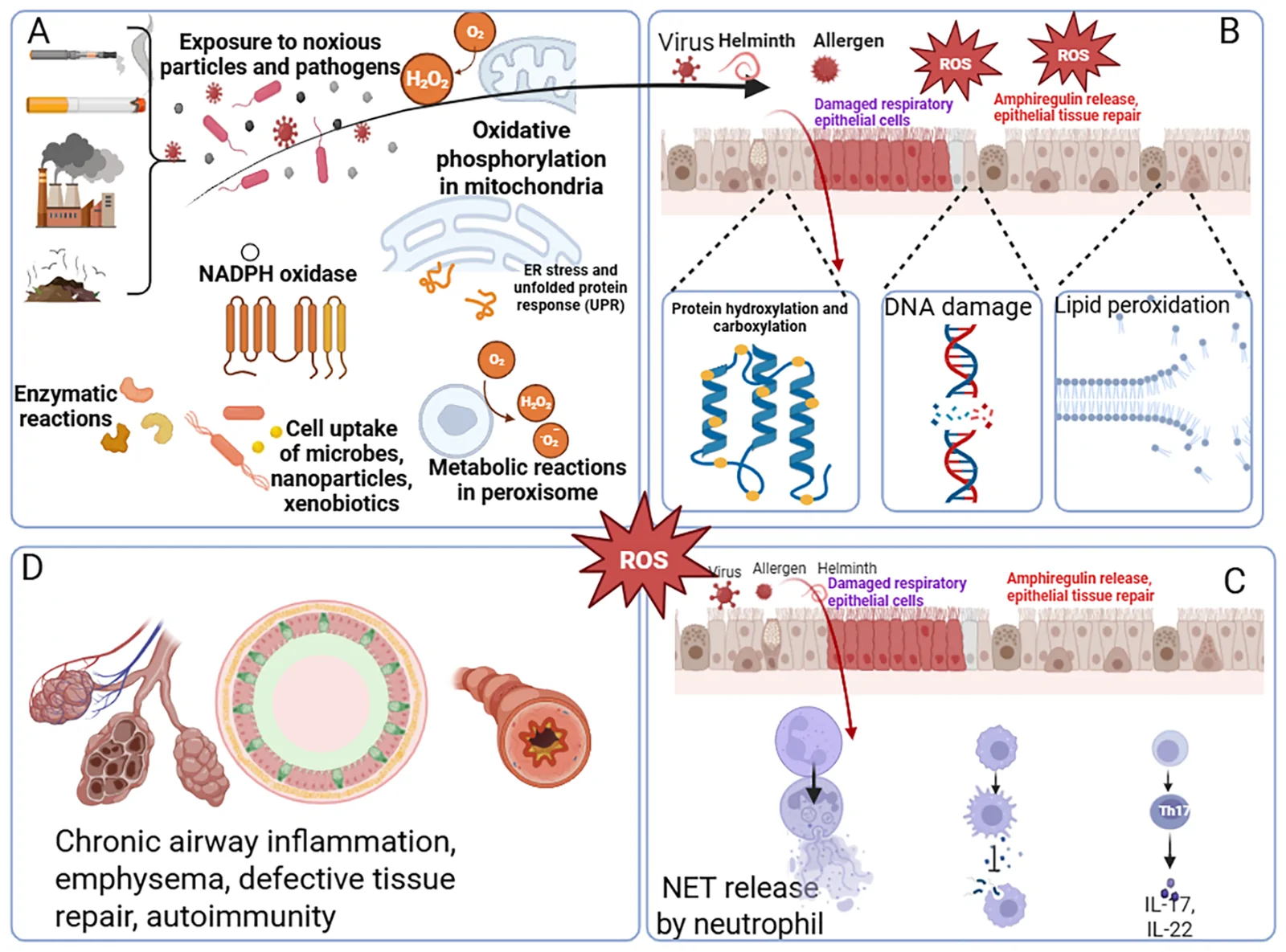
Central role of oxidative stress in COPD pathogenesis. **(A)** Sources of reactive oxygen and nitrogen species (ROS/RNS). Exogenous triggers include cigarette smoke, air pollution, and biomass fuel. Endogenous producers include the mitochondrial electron transport chain (ETC), NADPH oxidase (NOX) enzymes, and xanthine oxidase. **(B)** Molecular targets of ROS/RNS. ROS cause lipid peroxidation, protein carbonylation/nitration, and DNA damage (8-oxo-dG). **(C)** Consequences for key immune cell types. In neutrophils, ROS drive NETosis; in macrophages, ROS promote M1-like polarization and impair efferocytosis; in T cells, ROS favor Th17 differentiation and reduce regulatory T cell (Treg) stability. **(D)** Immunological outcomes. These changes result in chronic airway inflammation, tissue destruction (emphysema), defective resolution of inflammation, and potential autoimmunity.

An integrative perspective is essential: although oxidative stress is ubiquitous in COPD, the downstream immune phenotype that emerges depends on a complex context of cell-intrinsic programs, prior exposures, genetic predispositions, and—crucially—the epigenetic landscape that conditions gene responsiveness (**Figure 1**).

## 3 Epigenetic machinery in immune cells: DNA methylation, histone modifications, and noncoding RNAs

Epigenetic regulation establishes and maintains immune cell identity and functional plasticity (87). In COPD, chronic smoke exposure reshapes the epigenome in immune cells, creating durable, cell-type-specific alterations (88). These modifications determine cytokine repertoires, phagocytic competence, antigen presentation, and resolution programs. In this section we synthesize current evidence on DNA methylation dynamics, histone acetylation/deacetylation and other acylations, and non-coding RNA networks in key immune populations.

### 3.1 DNA methylation dynamics

DNA methylation at cytosine (5-mC) within CpG dinucleotides is catalyzed by DNMT1 (maintenance) and DNMT3A/3B (*de novo*) and erased by TET dioxygenases (89). Both DNMT and TET activity depend on metabolism and redox state: DNMTs use S-adenosylmethionine (SAM) as methyl donor, while TET enzymes require Fe(II) and α-KG (50). Oxidative stress influences both classes—ROS/NF-κB signaling increases DNMT expression, while oxidation of Fe(II) or depletion of α-KG inhibits TETs—shifting methylation balance toward hypermethylation (90).

In COPD alveolar macrophages, genome-wide bisulfite sequencing reveals selective hypomethylation of pro-inflammatory promoters (*IL6, TNF, MMP9*) and relative hypermethylation of antioxidant and mitochondrial loci (*BCL2, SOD2, TFAM*) (91). This composite rewiring stabilizes a pro-inflammatory, low-resolution phenotype. Mechanistically, cigarette smoke upregulates DNMT1 and DNMT3A via NF-κB and AP-1 while impairing TET activity through Fe(II)/α-KG disruption (28). Peng and colleagues described hypermethylation of the TFAM promoter after smoke exposure, resulting in mitochondrial dysfunction and increased mitochondrial ROS—a self-amplifying loop (92). Functionally, promoter hypomethylation at IL6 and MMP9 increases chromatin accessibility and transcription factor binding, potentiating cytokine release and matrix degradation (93).

In neutrophils, altered DNA methylation at *PADI4* and *ELANE* regulatory regions associated with increased NETosis propensity and higher exacerbation frequency (94). In T cells, the FOXP3 Treg-specific demethylated region (TSDR) shows reduced demethylation in some COPD studies, consistent with fewer stable Tregs (95). However, other studies find TSDR demethylation similar to controls but with functional impairment—suggesting interplay between DNA methylation, histone marks, and non-coding RNAs.

### 3.2 Histone acetylation and deacetylation

Histone acetylation at H3K27ac and H3K9ac is associated with open chromatin, added by histone acetyltransferases (HATs) including p300/CBP and GCN5, which use acetyl-CoA as donor (96). Histone deacetylases (HDACs) remove acetyl groups, with class I/II HDACs and the NAD^+^-dependent sirtuins (SIRT1-7) shaping chromatin compaction (97).

One of the most robust epigenetic alterations in COPD is reduced in HDAC2 expression and activity across alveolar macrophages, bronchial epithelial cells, and PBMCs (98). ROS mediate carbonylation and nitration of HDAC2, tagging it for ubiquitination and proteasomal degradation (99). The consequence is sustained hyperacetylation at inflammatory gene promoters and decreased repression of NF-κB-driven transcription. Loss of HDAC2 is tightly linked to corticosteroid resistance: glucocorticoid receptor (GR) recruitment requires HDAC2 to deacetylate histones and suppress transcription (100, 101). Sirtuins is also reduced in COPD lungs, partly due to oxidative modifications and decreased NAD^+^ availability (102, 103). Loss of SIRT1 contributes to hyperacetylation of NF-κB p65 and enhanced inflammatory. Restoration of NAD^+^ with nicotinamide riboside represents a potential strategy to rebalance acetylation states (104). Emerging histone acylations—including lactylation (driven by elevated lactate), succinylation and crotonylation—directly link metabolism to chromatin regulation (105–107). In COPD, heightened glycolysis with lactate accumulation could lead to histone lactylation, though this remains largely unexplored (108–110).

### 3.3 Noncoding RNA networks: miRNAs, lncRNAs, and circRNAs

Non-coding RNAs fine-tune gene expression at transcriptional and post-transcriptional levels (111). miR-21 is consistently upregulated in COPD, induced by ROS and NF-κB (112). miR-21 targets *SATB1* and S100A9, promoting neutrophilic inflammation (113). miR-146a is often downregulated in severe COPD, removing a brake on inflammation, whereas miR-155 is variably upregulated (114). The miR-29 family represses DNMT3A/B, and reduced miR-29 may permit DNMT upregulation and pathological methylation (115, 116).

**Figure 2:**
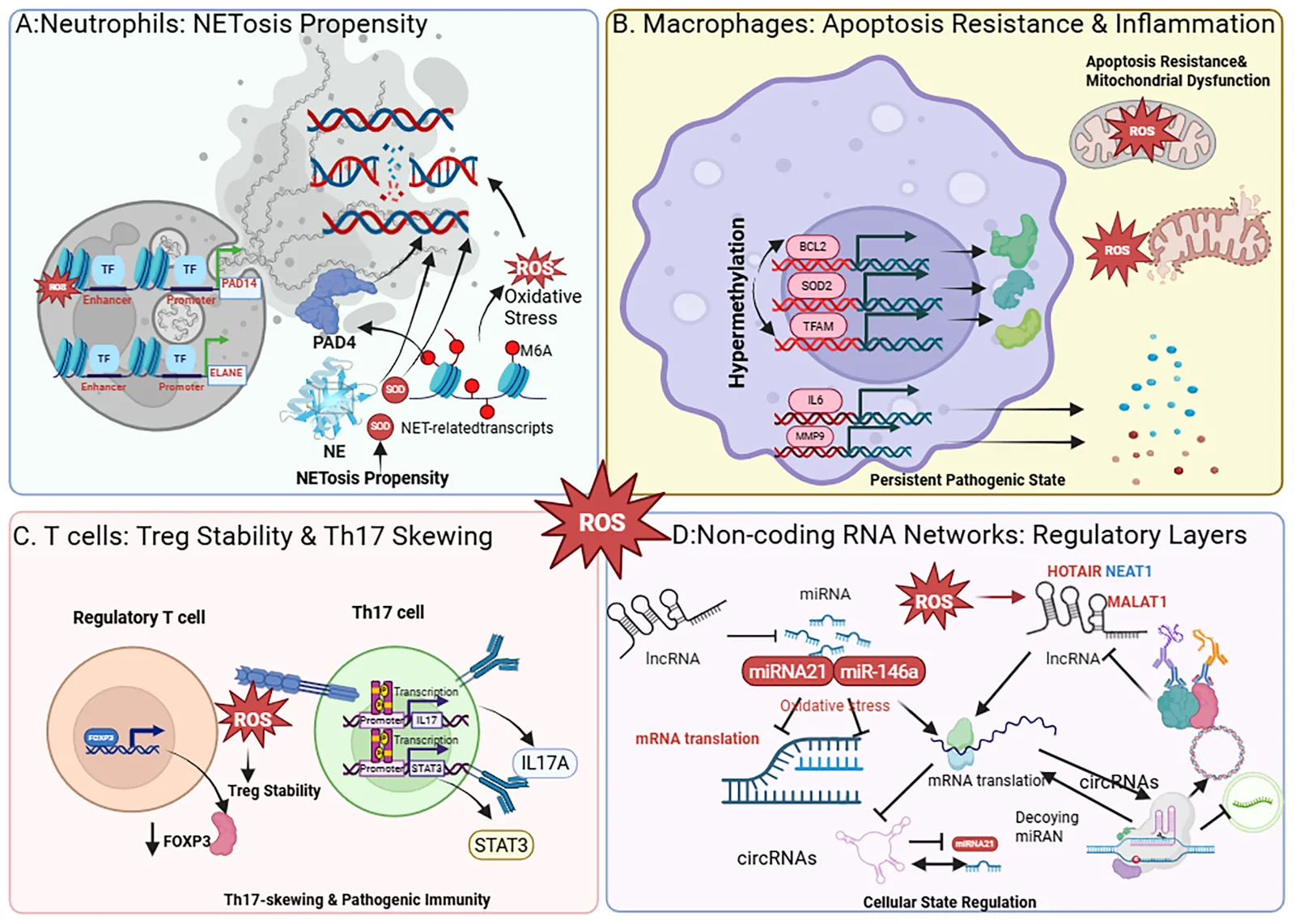
Cell type–specific epigenetic reprogramming in COPD immune cells. **(A)** Neutrophils. Hypomethylation at the PADI4 and ELANE promoters increases NETosis propensity. Altered m^6^A modification of NET-related transcripts may further regulate PAD4 expression. **(B)** Macrophages. Hypermethylation of BCL2, SOD2, and TFAM promotes apoptosis resistance and mitochondrial dysfunction, whereas hypomethylation of IL6 and MMP9 sustains pro-inflammatory cytokine release. Downregulation of HDAC2 enhances NF-κB-driven inflammation. **(C)** T cells. Hypermethylation (or reduced demethylation) of the FOXP3 TSDR impairs Treg stability. Permissive histone marks at IL17A and STAT3 support Th17 skewing. **(D)** Non-coding RNA networks. miRNAs (miR-21, miR-146a), lncRNAs (HOTAIR, NEAT1, MALAT1), and circRNAs add further regulatory layers. These epigenetic changes lock immune cells into persistent pathogenic states that are resistant to conventional therapy.

HOTAIR recruits DNMT1 to the *BCL2* promoter, increasing methylation and promoting endothelial apoptosis (117). NEAT1 enhanced pro-inflammatory cytokine production, while MALAT1 influences T cell activation (118–120). Circular RNAs are less studied, but early studies indicate differential circRNA expression in COPD (120–122). Non-coding RNAs both respond to and regulate epigenetic states, creating multi-layer regulatory networks that stabilize pathological phenotypes (**Figure 2**).

### 3.4 Critical gaps and future directions

**Figure 3:**
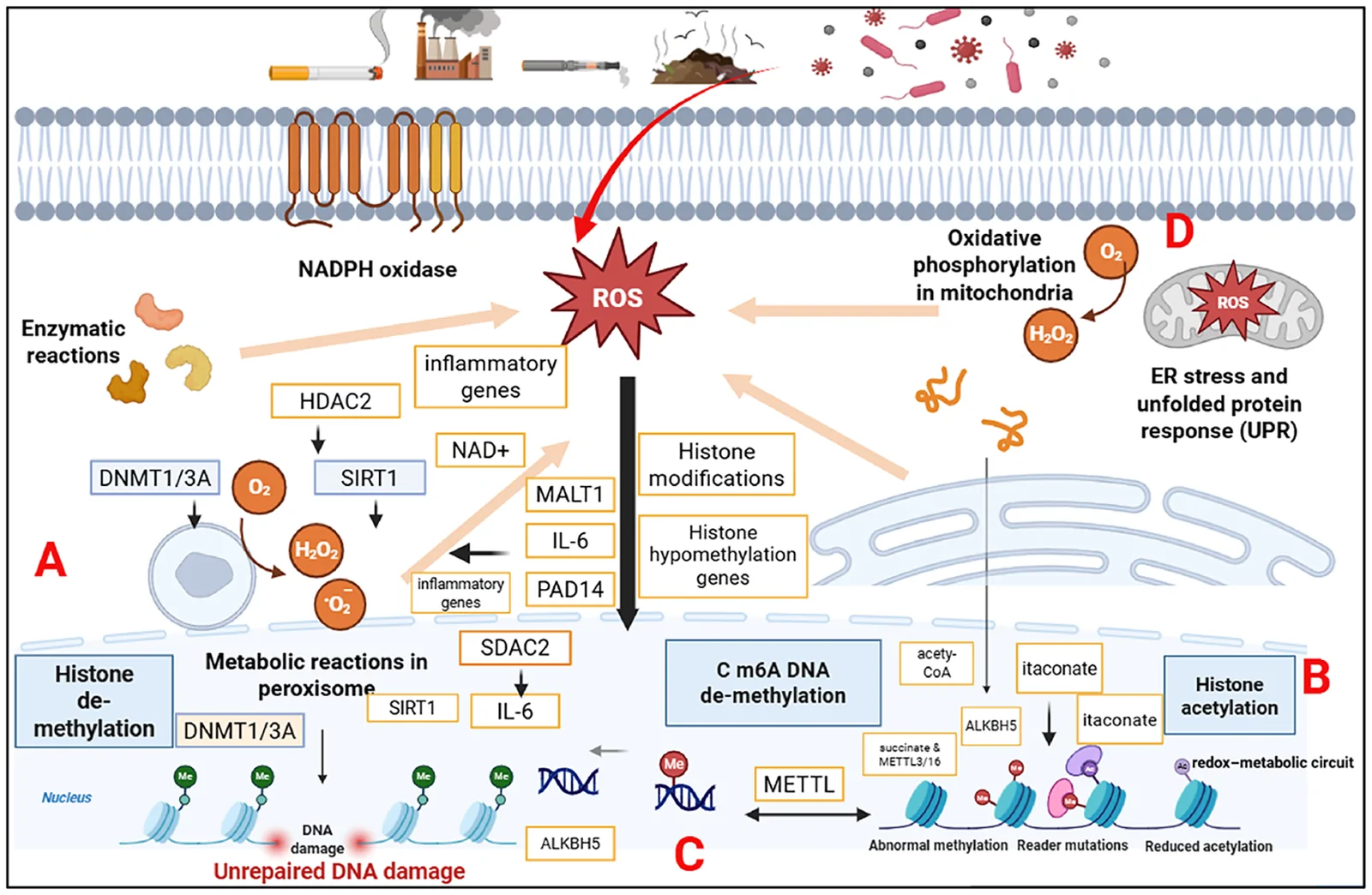
Molecular mechanisms by which ROS and metabolites drive epigenetic and epitranscriptomic modifications. The image details how oxidative stress and metabolic shifts regulate the activity of key enzymes. **(A)** DNA methylation: ROS/NF-κB upregulate DNMT1/3A, while oxidizing Fe²^+^ and depleting α-KG inhibit TET dioxygenases, leading to net DNA hypermethylation at specific loci. **(B)** Histone modifications: ROS cause HDAC2 carbonylation/nitration, promoting proteasomal degradation and resulting in histone hyperacetylation at inflammatory genes. SIRT1 is also inactivated via NAD^+^ depletion. **(C)** m^6^A RNA methylation: The demethylases FTO and ALKBH5 are Fe(II)/α-KG–dependent; ROS oxidize Fe²^+^ to Fe³^+^ and succinate accumulation (from TCA truncation) competitively inhibits these enzymes, causing m^6^A hypermethylation of transcripts such as *MALT1*, *IL6*, and *PADI4*. Writers (METTL3/16) depend on SAM, which is depleted under oxidative stress due to glutathione synthesis. **(D)** Metabolic-epigenetic loops: Metabolites (acetyl-CoA, NAD^+^, succinate, itaconate) act as cofactors or inhibitors, establishing self-perpetuating circuits. The figure integrates redox, metabolic, and epitranscriptomic layers into a unified mechanistic framework.

Despite substantial progress, several unresolved questions remain. First, most epigenetic studies use bulk tissue. single-cell multi-omic approaches are urgently needed (123, 124). Second, functional validation of methylation changes using epigenetic editing tools (dCas9-TET1, dCas9-DNMT3A) is lacking. Third, the roles of novel histone acylations in COPD remain unexplored. Addressing these gaps will move us from descriptive epigenomics to targeted interventions. Molecular mechanisms by which ROS and metabolites drive epigenetic modifications are illustrated in **Figure 3**.

## 4 m^6^A RNA methylation as a redox-sensitive epitranscriptomic regulator in COPD

### 4.1 The m^6^A machinery and its redox/metabolic sensitivity

m^6^A is a prevalent, reversible modification on eukaryotic mRNA that influences splicing, nuclear export, stability, localization, and translation (125). In immune cells, m^6^A modulates differentiation, cytokine output, and effector functions (126). Importantly, the catalytic machinery is biochemically linked to cellular metabolic and redox states: demethylases FTO and ALKBH5 are α-KG/Fe(II)-dependent dioxygenases, and writer activity depends on SAM supply (127–129).

The methyltransferase complex consists of METTL3 (catalytic subunit), METTL14 (RNA-binding structural partner) and regulatory subunits including WTAP, VIRMA, RBM15/15B and ZC3H13 (130). Two erasers, FTO and ALKBH5, reverse m^6^A via oxidative demethylation (131). Readers include YTH domain proteins (YTHDF1-3, YTHDC1-2) and IGF2BP family members, which determine functional outcome—YTHDF2 promotes decay, YTHDF1 promotes translation, and IGF2BP stabilize transcripts (132).

Critically for COPD, both erasers and writers are sensitive to redox state. FTO and ALKBH5 require reduced Fe(II) and α-KG; ROS can oxidize Fe²^+^ to Fe³^+^, impairing demethylase activity and causing m^6^A accumulation (33, 133). Under oxidative conditions in COPD lungs, suppression of FTO/ALKBH5 favors hypermethylation of mRNAs encoding inflammatory mediators and NETosis regulators (33, 134–136). SAM availability also affects writer activity. Under oxidative stress, cells divert methionine toward glutathione synthesis, diminishing SAM pools and impairing METTL3/METTL14 function (137). The α-KG/succinate ratio is another critical node. In inflammatory macrophages, succinate accumulates due to TCA cycle truncation and competitively inhibits ALKBH5 and FTO (138, 139). Itaconate, induced in activated macrophages, can alkylate and inhibit ALKBH5 (140–142). These mechanisms directly link macrophage metabolic reprogramming to epitranscriptomic control. 

### 4.2 m^6^A targets relevant to COPD

Multiple transcripts central to COPD immunopathology are regulated by m^6^A (143). *MALT1* (mucosa-associated lymphoid tissue lymphoma translocation protein 1) amplifies NF-κB signaling. Recent data indicate that METTL16-mediated methylation of *MALT1* mRNA, with subsequent stabilization by IGF2BP2, increases transcript stability and potentiates NF-κB pathways (144, 145). In COPD, this could sustain chronic NF-κB activation and steroid resistance (144). *IL6, IL17A* and *STAT3* transcripts are m^6^A-modified in various inflammatory contexts (146–148). IGF2BP readers can stabilize transcripts critical for Th17 differentiation. Andi Lin and colleagues provided direct evidence that METTL3/IGF2BP2-mediated m^6^A drives alveolar macrophage-dependent neutrophil recruitment by stabilizing CXCL8 and ICAM-1 transcripts in a COPD model (147). *PADI4* is a central executor of NETosis. Although direct evidence in COPD is limited, FTO knockdown has been reported to reduce NETosis in some models, suggesting FTO-mediated m^6^A removal may influence PADI4 expression (131, 149). *TFAM* and *SOD2* are putative m^6^A targets based on their roles in mitochondrial biogenesis and antioxidant defense. Hu and colleagues presented the m^6^A-methylomic landscape of lung tissues from COPD mice, revealing 430 genes with increased m^6^A and 3,995 with decreased modification, many related to immunity (150). Human cell-type-specific mapping is needed. Key m6A target genes implicated in COPD immunopathology are summarized in **Table 1**.

**Table 1:** Key m^6^A targets in COPD immunopathology.

Target gene	m^6^A regulation	Reader	Functional consequence in COPD	Evidence level	References
*MALT1*	Increased via METTL16	IGF2BP2	Sustained NF-κB activation, steroid resistance	Preclinical	(144)
*PADI4*	Context-dependent	YTHDF2/IGF2BP	Modulates NETosis (effect depends on reader balance)	*In vitro*	(151)
*IL6, STAT3, IL17A*	Increased in Th17 conditions	IGF2BP	Stabilisation of transcripts, Th17 skewing	Indirect	(152)
*TFAM, SOD2*	Putative (not well studied)	Unknown	Mitochondrial biogenesis, antioxidant defence	Hypothesised	(150)

### 4.3 Critical perspectives and therapeutic opportunities

m^6^A provides a rapid, reversible layer of regulation that can be tuned without permanently altering the genome (126). In COPD—where persistent epigenetic scarring limits responsiveness to conventional therapies—modulating m^6^A offers a way to reprogram post-transcriptional control (33, 151, 152).

Several small molecules have been developed to target the m^6^A machinery (147). FTO inhibitors such as meclofenamic acid derivatives, FB23, and FB23-2 inhibit FTO catalytic activity (153–155). FTO deficiency enhances Th17 differentiation through the CerS6-sphingolipid axis (156), while pharmacological FTO inhibition can suppress pro-inflammatory cytokine production (157). However, systemic FTO inhibition raises safety concerns: FTO is expressed in the central nervous system and plays roles in neurogenesis, learning, and memory (158). ALKBH5 inhibitors are under development but less advanced (131, 159); selective targeting in immune cells could modulate splicing and export of inflammatory transcripts (159). METTL3 inhibitors are being developed in oncology (160, 161); partial modulation may rebalance m^6^A rather than abolish it (161). Targeting m^6^A readers, such as blocking IGF2BP-mediated stabilization of pro-inflammatory mRNAs, offers a means to alter transcript fate without disturbing global methylation (134).

Delivery strategies and cell specificity are critical for translating m^6^A modulation into therapy. Inhaled formulations, such as nebulized small molecules or nanoparticles, permit lung-targeted modulation of m^6^A machinery while minimizing systemic exposure (162). Antibody- or ligand-conjugated nanoparticles enable selective delivery to macrophages, neutrophils, or T cells (163, 164). Gene-based approaches (RNAi, ASOs, CRISPR-dCas13 epitranscriptomic editing) could directly modify specific transcripts (33, 147). Natural products have also been reported to modulate m^6^A machinery (165). Polyphenols such as baicalin, curcumin, and resveratrol can influence writer/eraser expression, redox state, or metabolite pools (166). The Shenqi Tiaoshen formula has been reported to reduce METTL16/IGF2BP2-mediated MALT1 m^6^A stabilization (167). While intriguing, such claims require independent replication and mechanistic validation.

## 5 Immunometabolism–epigenetics crosstalk: the molecular bridge

### 5.1 Metabolic reprogramming in COPD immune cells

Immune cell function is linked to cellular metabolism. Pro-inflammatory (M1) macrophages and effector T cells (notably Th17) engage aerobic glycolysis—the “Warburg” phenotype (168). Anti-inflammatory or reparative states, including M2 macrophages and Tregs, favor oxidative phosphorylation (OXPHOS) and fatty acid oxidation (169). In COPD, alveolar macrophages exhibit elevated glycolysis and impaired mitochondrial respiration (170, 171). T cells from COPD lungs show increased glycolytic signatures in effector populations and mitochondrial dysfunction in Tregs (172).

### 5.2 Metabolites as direct epigenetic cofactors

These metabolic shifts directly influence the pools of metabolites required for epigenetic enzyme activity, as summarized in **Table 2**. 

**Table 2:** Metabolites as epigenetic cofactors in COPD.

Metabolite	Epigenetic enzyme(s)	Effect of oxidative stress in COPD	Consequence	References
SAM	DNMTs, histone methyltransferases, METTL3/16	Depleted (diverted to glutathione synthesis)	Global DNA/RNA hypomethylation at some loci, but paradoxical hypermethylation at specific promoters?	(173)
Acetyl-CoA	HATs (p300/CBP)	Increased (via glycolysis)	Histone hyperacetylation at inflammatory loci	(174)
α-KG	TETs, KDMs, FTO, ALKBH5	Decreased (TCA truncation; oxidised to succinate)	Impaired demethylation → DNA hypermethylation, m^6^A accumulation	(175, 176)
NAD^+^	Sirtuins (SIRT1-7)	Decreased (oxidative consumption, PARP activation)	Reduced SIRT1 activity → hyperacetylation, inflammation	(177, 178)

### 5.3 Self-perpetuating epigenetic–metabolic loops

Crucially, epigenetic changes feedback to reinforce pathological metabolic. Hypermethylation of *TFAM* reduces mitochondrial biogenesis and increases mtROS, depleting α-KG (92, 179). Hypermethylation of *SOD2* diminishes antioxidant capacity, raising ROS levels (179). Thus, an initial oxidative insult can become self-perpetuating through epigenetic–metabolic loops. This explains why simple antioxidant therapy fails: it removes the trigger but not the epigenetic “scar” (180). Therefore, strategies must restore metabolic cofactor balance (e.g., NAD^+^ precursors, α-KG supplementation, succinate reduction) alongside epigenetic modulation (181).

## 6 NETosis and trained immunity: clinical consequences of oxidative-epigenetic reprogramming

### 6.1 NETosis as an oxidative-epigenetic event

Neutrophil extracellular trap formation (NETosis) is an antimicrobial mechanism that, when dysregulated, contributes to tissue injury and autoimmunity. Classical (suicidal) NETosis requires NOX2-derived ROS, which trigger calcium influx and PAD4-mediated histone citrullination (182). Epigenetic regulation of NETosis is well documented: in COPD neutrophils, hypomethylation at *PADI4* and *ELANE* regulatory regions correlates with increased transcriptional readiness (183). Although direct evidence for m^6^A modification of PADI4 in COPD is lacking, such regulation has been inferred from other inflammatory models (149). The effect of m^6^A on PADI4 expression is context-dependent and reader-specific, highlighting the need for cell type–specific studies.

### 6.2 Impaired resolution of inflammation

Specialized pro-resolving mediators (SPMs) such as lipoxins, resolvins, protectins, and maresins derive from enzymatic oxygenation of polyunsaturated fatty acids by lipoxygenases (184). Oxidative modification of lipoxygenases, altered substrate availability, and epigenetic silencing of SPM biosynthetic genes reduce SPM production in COPD (185). Diminished SPM levels impair efferocytosis and resolution signaling, allowing NETosis to continue unchecked. NETs themselves exacerbate impaired resolution: neutrophil proteases degrade lipid mediators, including lipoxin A^4^ and resolvin D1 (55). Extracellular chromatin hinders macrophage recognition of apoptotic cells, delaying resolution. Moreover, NETs expose modified self-antigens (oxidized, citrullinated proteins) that can be processed and presented by DCs, contributing to autoimmune-like responses (57).

### 6.3 Therapeutic targeting of NETosis

Several natural compounds have shown potential in modulating NETosis (186, 187). Berberine exhibits PAD4 inhibitory activity, reducing NET formation (188, 189). Baicalin possesses elastase inhibitory and antioxidative properties (190–192). Curcumin influences HDAC activity and NF-κB signaling while scavenging ROS (193, 194). However, most studies were conducted in non-COPD models and pharmacokinetic data in the respiratory tract are lacking. Potential combination strategies include pairing targeted antioxidants with HDAC2 activators (low-dose theophylline) (195), or combining PAD4 inhibition with SPM analogs (195). Inhaled formulations may achieve high local concentrations while minimizing systemic effects. Translational challenges include balancing NETosis suppression with preserved antimicrobial defense (196). Identifying patients with NETosis-predominant endotypes (high sputum citH3, NE-DNA, low SPMs) will be crucial.

### 6.4 Trained immunity: epigenetic-metabolic memory in COPD

Trained immunity is a newly recognized form of innate immune memory in which monocytes, macrophages, and natural killer cells exhibit enhanced responses to secondary stimuli following an initial challenge (197). Unlike adaptive immunity, which relies on antigen-specific receptors, trained immunity is mediated by sustained metabolic and epigenetic reprogramming (198).

The mechanisms of trained immunity are highly relevant to COPD. β-glucan and BCG induce trained immunity through increased glycolysis and glutaminolysis (metabolic reprogramming) and sustained H3K4me3 at promoters of inflammatory genes (epigenetic reprogramming) (199). These changes are mediated by the Akt-mTOR-HIF-1α pathway and depend on fumarate accumulation, which inhibits KDM5 histone demethylases (199). In COPD, the airway microenvironment—characterized by chronic oxidative stress, persistent microbial colonization, and repeated inflammatory insults—is ideally suited to induce trained immunity in lung macrophages (200). Smoke exposure itself may act as a training stimulus. Cigarette smoke-trained macrophages show enhanced IL-6 and TNF-α production upon LPS rechallenge, mediated by DNMT and HDAC changes (197). Similarly, bacterial components (LPS, flagellin) in COPD airways can induce trained immunity, potentially contributing to the heightened inflammatory responses characteristic of exacerbations (201).

Several unanswered questions merit investigation. Does trained immunity in alveolar macrophages contribute to the “memory” of exacerbations, making patients progressively more susceptible? Can trained immunity be therapeutically targeted—for example, by inhibiting metabolic or epigenetic pathways that sustain it? Are there COPD endotypes characterized by heightened trained immunity, and could this serve as a biomarker for exacerbation risk? Addressing these questions will require longitudinal sampling of airway macrophages from COPD patients before and after exacerbations, combined with epigenetic and metabolic profiling.

## 7 From bench to bedside: mechanistic patterns, biomarkers, and treatable traits

The heterogeneity of COPD demands refined approaches that goes beyond spirometry and symptom scores to include mechanistic classifiers amenable to targeted interventions. An “epigenetic-oxidative” framework integrates oxidative burden, epigenetic state, immunometabolism, and functional biomarkers to define biologically coherent patterns that may eventually guide therapy.

### 7.1 Limitations of antioxidant monotherapies

Clinical experience with antioxidant agents in COPD has been disappointing (202). Trials of N-acetylcysteine and erdosteine show modest and inconsistent effects on exacerbation reduction (203). Antioxidant vitamins have not demonstrated robust efficacy (204, 205). Nrf2 pathway activators have shown limited clinical translation.

Mechanistic limitations are central. Antioxidants neutralize current oxidants but do not reverse oxidant-induced epigenetic modifications (206). Many antioxidants fail to reach relevant cellular microdomains (mitochondria, phagosomes). Broad suppression risks impairing physiological ROS-dependent host defense. Moreover, oxidative stress alters metabolism beyond oxidant burden—depleting NADPH, shifting TCA flux, altering α-KG, acetyl-CoA, and SAM. Removing oxidants does not instantly restore metabolic homeostasis. A comprehensive approach must combine redox modulation with interventions addressing epigenetic reprogramming. Potential strategies include HDAC2 activators, DNA methylation inhibitors, metabolic support (NAD^+^ precursors, α-KG), precision delivery of mitochondrial-targeted antioxidants, and CRISPR-based epigenetic editors.

### 7.2 Mechanistic patterns of epigenetic dysfunction: a research framework

Based on the mechanistic synthesis above, several candidate molecular patterns emerge that may contribute to COPD heterogeneity. These include a hypermethylation-prone pattern (characterized by DNMT upregulation and TET suppression, affecting genes such as BCL2, SOD2, and TFAM), an m^6^A-dysregulated pattern (featuring altered writer/eraser balance and accumulation of m^6^A on transcripts such as MALT1 and IL6), a NETosis-prone pattern (associated with PADI4/ELANE hypomethylation and low SPM levels), and an HDAC2-deficient pattern (linked to corticosteroid resistance). Each of these patterns has been associated with specific molecular and functional alterations in preclinical or translational studies, and each suggests distinct biomarker candidates—including methylated DNA, m^6^A-modified transcripts, NET remnants, and HDAC2 activity—that could be measured in blood or sputum to guide future investigation.

However, we must emphasize a critical limitation: these patterns currently represent mechanistic hypotheses rather than validated clinical endotypes. For any such pattern to qualify as a true endotype, it must demonstrate consistent associations with distinct clinical phenotypes (chronic bronchitis versus emphysema), specific immune response profiles (type 1, type 2, or type 3), differential treatment responses, and predictable clinical outcomes including exacerbation frequency and disease progression. These associations have not yet been established. We therefore propose these patterns as a conceptual framework to guide hypothesis-driven biomarker discovery and mechanistic investigation, rather than as a classification ready for clinical deployment. Future prospective studies integrating multi-omics data with deep clinical phenotyping are essential to determine whether these mechanistic patterns indeed define clinically meaningful patient subsets.

### 7.3 Biomarkers for patient stratification

Blood-based biomarkers offer a minimally invasive approach. Cell-free methylated DNA can be quantified by targeted bisulfite PCR. m^6^A-modified transcripts can be assessed using m^6^A-IP-qPCR. NET remnants (citH3, NE-DNA, MPO-DNA) can be measured by ELISA. Oxidative and metabolic markers include nitrotyrosine, 8-oxo-dG, GSH/GSSG, lactate, and NAD^+^/NADH ratios. Sputum and airway sampling provides more direct assessment. Single-cell epigenomics in sputum cells allows direct evaluation of airway immune cell epigenetic states. SPM levels (LXA^4^, RvD1) indicate resolution capacity. Local metabolomics can measure succinate, itaconate, and acetyl-CoA proxies. For feasibility, biomarker panels should be tiered: readily available blood assays for initial stratification, while complex sputum single-cell epigenomics can be reserved for specialized centers.

## 8 Future directions and unanswered questions

The intersection of oxidative stress, immunometabolism, epigenetics, and epitranscriptomics provides a unifying conceptual framework to explain COPD heterogeneity and therapeutic resistance. Translating this integrated framework into clinical practice will require progress along several interrelated fronts.

First, single-cell and spatial multi-omics approaches are urgently needed to dissect the cellular and spatial heterogeneity of epigenetic reprogramming in COPD. Integrating scRNA-seq, scATAC-seq, methylome, and m^6^A mapping in matched airway and blood immune cells from well-phenotyped COPD cohorts will reveal cell type–specific programs and identify the dominant pathogenic subsets (207). Spatial epigenomics, applied to lung tissue microenvironments, will map how epigenetic and metabolic states vary across peribronchiolar niches and regions of high NET burden, providing insight into how local oxidative niches shape cell behavior and structural remodeling (208). Longitudinal studies with sampling surrounding exacerbations will determine whether epigenetic and epitranscriptomic shifts precede clinical worsening and whether they are reversible with targeted intervention (209).

Second, the development of epigenetic and epitranscriptomic editing tools represents an ambitious but transformative goal. Locus-specific editors such as dCas9–TET1 or dCas9–DNMT3A for DNA methylation, and CRISPR–dCas13-based systems for RNA methylation, could correct pathogenic marks in a targeted manner (210, 211). Realizing this potential will require parallel mechanistic studies to detail how ROS and metabolites—including succinate, itaconate, and SAM depletion—alter the catalytic activity, structure, and localization of DNMTs, TETs, HDACs, FTO/ALKBH5, and m^6^A writers and readers (139, 212). Such tools would not only enable causal testing of whether specific epigenetic marks drive disease phenotypes but ultimately provide a route to targeted therapeutic correction.

Third, the integration of clinical, imaging, and multi-omics data using machine learning approaches offers a powerful strategy to identify novel endotypes and predict treatment response (213, 214). This effort will require large, well-annotated cohorts with longitudinal sampling, as well as robust computational pipelines capable of handling the complexity and heterogeneity of COPD data, with the ultimate goal of developing a clinically deployable decision-support tool for real-time biomarker-guided therapy selection.

Fourth, Traditional Chinese Medicine (TCM) formulations and natural compounds represent an interesting research avenue for exploring combination modulation of the oxidative-epigenetic axis. Various compounds have been reported to modulate ROS, HDAC2, m^6^A writers/erasers, and NETosis in preclinical models (144). For example, the Shenqi Tiaoshen formula has been suggested to modulate the METTL16-m^6^A-MALT1-NF-κB axis in COPD (144, 167). However, we emphasize that these findings are largely based on network pharmacology predictions and preclinical models, with limited pharmacokinetic and mechanistic validation. Rigorous identification of active components, detailed biochemical characterization, and well-controlled clinical trials are essential before TCM-derived interventions can be considered as evidence-based therapeutic options. TCM should be positioned as a source of lead compounds for mechanistic investigation, rather than as a proven therapeutic strategy, until more robust clinical evidence emerges.

Finally, clinical trial designs must evolve to test combined modality strategies that target both redox and epigenetic axes—for example, combining N-acetylcysteine with low-dose theophylline and a PAD4 inhibitor—in patients selected by biomarker panels. Given the pleiotropy of epigenetic and metabolic pathways, early-phase trials must incorporate intensive safety monitoring covering infection surveillance, metabolic panels, and assessment of off-target epigenetic effects. The parallel development of validated companion diagnostics, including blood methylation assays and m^6^A qPCR, will be essential for regulatory approval and clinical adoption.

## 9 Conclusion

Oxidative stress is not merely a damaging force but a powerful instructor that reprograms immune cells toward persistent pro-inflammatory, pro-NETotic, and resolution-deficient states. By integrating m^6^A epitranscriptomics, immunometabolism, and trained immunity, we now appreciate that the redox-epigenetic axis operates at multiple layers—from DNA and histones to RNA—and is intimately connected to cellular fuel selection. This understanding moves us beyond failed “one-size-fits-all” antioxidant trials toward mechanism-based, endotype-driven precision medicine. Substantial challenges remain: the complexity of epigenetic codes, the lack of validated biomarkers, and the need for rigorous, biomarker-guided clinical trials. Future research must prioritize single-cell multi-omic mapping, spatial epigenomics, and the development of inhaled, cell-selective delivery systems for epigenetic modulators. Only by deciphering the cell type–specific epigenetic codes that lock immune cells into pathogenic states—and by launching trials that match interventions to mechanistic endotypes—can we truly alter the trajectory of this devastating disease.

## Glossary

5-azacytidine: 5-azacytidine (DNA methyltransferase inhibitor)
5hmC: 5-hydroxymethylcytosine
5mC: 5-methylcytosine
8-OHdG: 8-hydroxy-2′-deoxyguanosine (also 8-oxo-dG)
Akt: protein kinase B
ALKBH5: AlkB homolog 5
ALOX5: arachidonate 5-lipoxygenase
ALOX15: arachidonate 15-lipoxygenase
AP-1: activator protein 1
ASOs: antisense oligonucleotides
circRNAs: circular RNAs
citH3: citrullinated histone H3
COPD: chronic obstructive pulmonary disease
CRISPR: clustered regularly interspaced short palindromic repeats
CXCL8: C-X-C motif chemokine ligand 8 (IL-8)
dCas13: dead Cas13
dCas9: dead Cas9
dCas9-DNMT3a: dead Cas9 fused to DNA methyltransferase 3A
dCas9-TET1: dead Cas9 fused to ten-eleven translocation 1
DNMT1: DNA methyltransferase 1
DNMT3A: DNA methyltransferase 3A
DNMT3B: DNA methyltransferase 3B
DNMTs: DNA methyltransferases
ELANE: elastase, neutrophil expressed
ERK: extracellular signal-regulated kinase
Fe2+: ferrous ion
Fe3+: ferric ion
FTO: fat mass and obesity-associated protein
GCN5: general control non-derepressible 5
GR: glucocorticoid receptor
GSH: reduced glutathione
GSSG: oxidized glutathione
H2O2: hydrogen peroxide
H3K27ac: histone H3 lysine 27 acetylation
H3K9ac: histone H3 lysine 9 acetylation
HATs: histone acetyltransferases
HDAC2: histone deacetylase 2
HIF-1α: hypoxia-inducible factor 1-alpha
HOTAIR: HOX transcript antisense RNA
ICAM-1: intercellular adhesion molecule 1
ICS: inhaled corticosteroids
IGF2BP1-3: insulin-like growth factor 2 mRNA-binding proteins 1-3
ILC2: type 2 innate lymphoid cell
ILC3: type 3 innate lymphoid cell
ILCs: innate lymphoid cells
lncRNAs: long non-coding RNAs
LXA4: lipoxin A₄
m⁶A: N⁶-methyladenosine
MALAT1: metastasis-associated lung adenocarcinoma transcript 1
MALT1: mucosa-associated lymphoid tissue lymphoma translocation protein 1
METTL14: methyltransferase-like 14
METTL16: methyltransferase-like 16
METTL3: methyltransferase-like 3
miRNAs: microRNAs
miR-146a: microRNA-146a
miR-155: microRNA-155
miR-21: microRNA-21
miR-29: microRNA-29
MitoQ: mitoquinone
MPO: myeloperoxidase
MPO-DNA: myeloperoxidase-DNA complexes
mRNA: messenger RNA
mtROS: mitochondrial reactive oxygen species
NAC: N-acetylcysteine
NAD+: nicotinamide adenine dinucleotide (oxidised form)
NADH: nicotinamide adenine dinucleotide (reduced form)
NEAT1: nuclear paraspeckle assembly transcript 1
NF-κB: nuclear factor kappa-light-chain-enhancer of activated B cells
NOX2: NADPH oxidase 2
NOX4: NADPH oxidase 4
O2-: superoxide anion
ONOO-: peroxynitrite
OXPHOS: oxidative phosphorylation
p300/CBP: p300/CREB-binding protein
PAD4: peptidylarginine deiminase 4 (also PADI4)
PADI4: peptidyl arginine deiminase 4 (gene)
PDE4: phosphodiesterase 4
PI3K: phosphoinositide 3-kinase
PM2.5: particulate matter with aerodynamic diameter ≤
qPCR: quantitative polymerase chain reaction
RBM15/15B: RNA-binding motif protein 15/15B
RNS: reactive nitrogen species
ROS: reactive oxygen species
RvD1: resolvin D1
SAM: S-adenosylmethionine
SATB1: special AT-rich sequence-binding protein 1
SIRT1-7: sirtuins 1-7
SOD2: superoxide dismutase 2
SPMs: specialized pro-resolving mediators
STAT3: signal transducer and activator of transcription 3
TCA: tricarboxylic acid (Krebs cycle)
TCM: Traditional Chinese Medicine
TET: ten-eleven translocation (enzyme family)
TET1-3: ten-eleven translocation 1-3
TFAM: mitochondrial transcription factor A;Th17, T helper 17 cells
Treg: regulatory T cells
TSDR: Treg-specific demethylated region
VIRMA: vir-like m6A methyltransferase associated
WTAP: Wilms tumour 1-associated protein
YTHDC1-2: YTH domain containing 1-2
ZC3H13: zinc finger CCCH-type containing 13.
Target gene: m⁶A regulation*MALT1*, Increased via METTL16
*PADI4*: Context-dependent
IL6: *STAT3, IL17A*, Increased in Th17 conditions
TFAM: *SOD2*, Putative (not well studied)
